# Studying the Gene Expression of *Penicillium rubens* Under the Effect of Eight Essential Oils

**DOI:** 10.3390/antibiotics9060343

**Published:** 2020-06-19

**Authors:** Zuzana Kisová, Andrea Šoltýsová, Mária Bučková, Gábor Beke, Andrea Puškárová, Domenico Pangallo

**Affiliations:** 1 Institute of Molecular Biology, Slovak Academy of Sciences, Dúbravská cesta 21, 84551 Bratislava, Slovakia; zuzana.kisova@savba.sk (Z.K.); maria.buckova@savba.sk (M.B.); gabor.beke@savba.sk (G.B.); andrea.puskarova@savba.sk (A.P.); 2 Department of Molecular Biology, Faculty of Natural Sciences of Comenius University, Ilkovičova 6, 84215 Bratislava, Slovakia; andrea.soltysova@uniba.sk; 3 Institute for Clinical and Translational Research, Biomedical Research Center, Slovak Academy of Sciences, Dúbravská cesta 9, 84505 Bratislava, Slovakia

**Keywords:** *Penicillium rubens*, essential oils, growth inhibition, RNA microarray, gene expression, metabolic pathway analysis

## Abstract

Essential oils (EOs) are well-known for their beneficial properties against a broad range of microorganisms. For the better understanding of their mechanism of action in fungi, a microarray approach was used in order to evaluate the gene expression of *Penicillium chrysogenum* (recently renamed *P. rubens*) exposed to the indirect contact (vapors) of eight EOs. The selection of assayed EOs was based on their antifungal activity. The extraction of RNA and the microarray hybridization procedure were optimized for the analysis of *P. rubens.* Gene ontology annotation was performed to investigate the functional analysis of the genes. To uncover the metabolic pathway of these differentially expressed genes, they were mapped into the KEGG BRITE pathway database. The transcriptomic analysis showed that, from a total of 12,675 genes, only 551 genes are annotated, and the other 12,124 genes encoded hypothetical proteins. Further bioinformatic analysis demonstrated that 1350 genes were upregulated and 765 downregulated at least with half (four) of the utilizing EOs. A microarray investigation has confirmed the main impact of EOs to metabolic processes in *P. rubens* involved in vital functions. Presumably, this is the first time that a microarray hybridization analysis was performed in order to evaluate the gene expression of *P. rubens* exposed to various EOs.

## 1. Introduction

Nowadays, when antimicrobial resistance becomes a worldwide problem [1], the alternative ways of treatment, such as the use of natural products, are getting the foreground [2]. EOs are synthetized via the secondary metabolism from different parts of plants, such as buds, leaves, flowers, twigs, seeds, fruits, roots, bark or wood, located in secretory tissues [3] (p. 95). From a chemical point of view, EOs are a complex mixture of volatile elements, mainly terpenoids, phenol-derived aromatic compounds and aliphatic compounds, with high antibacterial, antifungal, antiviral, antiparasitical and insecticidal activities [4].

The use of spices and herbs in food preservation or in medicine for their health benefits has a long history [5]. The botanical family *Lamiaceae* has significant representatives for the production of EOs, such as *Origanum vulgare* L., *Mentha piperita* L. and *Thymus vulgaris* L. with a large-scale of antioxidant and antimicrobial characteristics [6]. Tea tree EOs from *Melaleuca alternifolia* Maiden and Betche (*Myrtaceae*) have shown antiseptic and anti-inflammatory effects [7] and medical plant *Eugenia caryophyllata* Thunb., which belongs also to the family *Myrtaceae,* is known for its antifungal, antiviral, antioxidant, anti-inflammatory and anticancer attributes [8]. Previous studies have shown diverse effects of cinnamon plants (*Cinnamomum cassia* L.) of the *Lauraceae* family: antidiabetic, antioxidant, anti-inflammatory, antimicrobial, anticancer and other important activities, such as lipid or cardiovascular disease-lowering compounds [9]. *Cymbopogon flexuosus* Nees ex Steud., worldwide known as lemongrass (from East Indian), belongs to the *Poaceae* family, which is important in EO productions [10,11], possessing antifungal and antimicrobial properties [12,13]. EOs obtained from *Thuja plicata* Donn. (*Cupressaceae*) tree leaves were assigned a broad spectrum of antimicrobial abilities, as well as potential uses in the reduction of sick building syndrome (SBS) [14].

Airborne microorganisms, such as fungi and their spores, are widespread in outdoor and indoor habitats, too [15]. From a health point of view, fungi are representatives of serious health hazards, including respiratory problems, fungal infections (mycoses), irritant effects, allergy reactions and other nonspecific medical troubles [16,17], and could also be a potential problem linked with sick building syndrome—SBS [17,18]. Members of the genus *Penicillium* are considered to be among these colonizers, with diverse roles: decomposition of organic materials by its enzymatic properties, producing toxic secondary metabolites (SM; mycotoxins) and even indoor air quality contaminators [19]. Filamentous fungus *Penicillium rubens* was, according to the study of Wilson and Straus [20], the most detected species associated with SBS.

The fungal contamination in an indoor environment can be solved using fungicides able to kill or inhibit the fungal growth [16]. Synthetic antifungal agents such as bleach, alcohol (100%), quaternary ammonium compounds, formaldehyde and multipurpose industrial disinfectants such as Cavicide^®^ and Virkon^®^ are detergents frequently used for contamination removal [16], for their unpleasant effects on human health are considered as hazardous, which can cause respiratory tract irritation and allergic respiratory and skin reactions. The demand of natural fungicides instead of synthetic chemicals is rising [21] due to concerns regarding human health [22,23]. Replacing synthetic antifungal agents with plant extracts could be an alternative that can reduce, remove or control fungi and molds in a specific environment [23].

Many studies are present in the literature about the effects of EOs against microorganisms [24], but there is little information, especially about fungi, regarding the genetic and metabolism mechanisms that cause these antimicrobial effects. Several authors have described the fungicide activity of EOs as that their components cause the disintegration of fungal hyphae, amplifying membrane permeability [25,26]. An antifungal effect is associated with the lipophilicity of EOs that allows penetration through cell walls and the manipulation with enzymes implicated in the synthesis of cell walls, thus making changes in the morphological characteristics of the fungus [26,27]. In order to extend our knowledge, a key research is to understand the mechanisms of action of EOs to molds. A microarray hybridization approach is a useful method that enables the simultaneous detection of the expression levels of thousands of genes [28]. The expression of an organism’s genes is affected by environmental growth conditions [29] and serves to provide information about gene regulation [30]. In this study, we have investigated the fungicide effects of the vapor of eight EOs on the gene expression of *P. rubens* using a microarray strategy.

## 2. Results

### 2.1. Inhibition of Mycelial Growth Using EOs Vapors

In present study the inhibition concentrations of eight EOs vapors, for which the microscopic filamentous fungus still survived, were investigated. The microarray hybridizations were carried out with the RNA obtained from the mycelia of *P. rubens* isolated 24 h after exposure *to Cymbopogon flexuosus* (CF), *Cinnamomum cassia* (CC), *Origanum vulgare* (OV), *Thymus vulgaris* (TV) and *Thuja plicata* (TP) EO vapors and 48 h after exposure to *Mentha piperita* (MP), *Eugenia caryophyllata* (EC) and *Melaleuca alternifolia* (MA) EO vapors. The incubation time was dependent on the inhibition ability of EOs to the mycelial growth. In the end of the exponential phase, the samples were collected. A concentration of selected EOs was in the range of 0.01–0.05 µg/mL. Under these conditions, the conidia formation and germination of spores was suppressed and coupled with the activation of stress-responding processes. The RNA extracted from the fungus without exposure to EOs, after 24 and 48 h of cultivation, served as the control.

### 2.2. Impact of Essential Oils on Genome-Wide Gene Expression

The gene expression profile of *P. rubens* was evaluated by Gene Expression 8 × 15K custom Microarray Slide containing 12,675 genes, from which only 551 are annotated, and the other 12,124 genes encode hypothetical proteins. A microarray-based analysis allowed us to reveal differentially expressed genes (fold change >2) by comparing the expression profiles of genes between the control and the fungus exposed to EOs.

We identified several differently expressed genes (Table 1) for targeted EOs, of which 1430 upregulated genes and 833 downregulated genes were detected in at least four assayed EOs (*n* ≥ 4). However, from 2263 genes (1430 upregulated and 833 downregulated), 148 (80 upregulated and 68 downregulated) genes were with mixed regulation, i.e., genes were not just up- or downregulated by at least four EOs, but also, in at least one sample, they were regulated in the other way (e.g., one gene upregulated by four EOs and downregulated by one EO). These genes were discarded from further analysis. The final number of genes, which had the same regulation in at least half of the samples exposed to different EOs (*n* ≥ 4): 1350 upregulated and 765 downregulated genes (Table S1).

### 2.3. Gene Ontology Analysis

**Figure 1:**
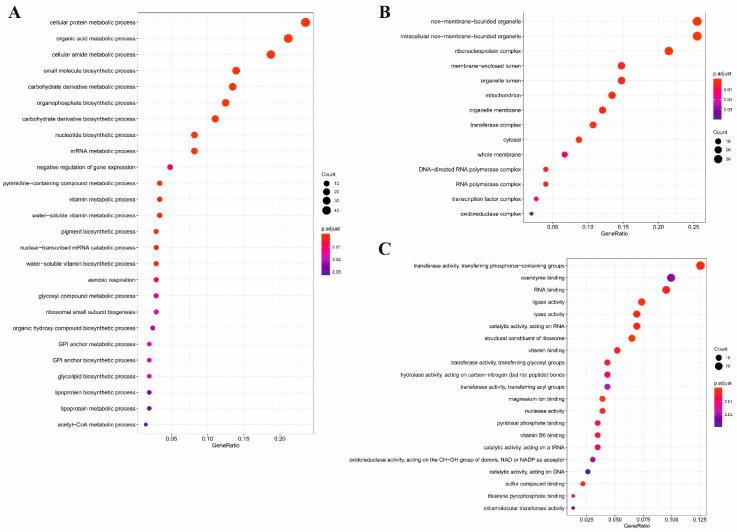
Results of a gene ontology (GO) gene set enrichment analysis (GSEA) of *Penicillium* *rubens* upregulated genes for different GO domains’ (**A**) biological processes, (**B**) cellular components and (**C**) molecular functions. The figures describe the rate of involvement of upregulated genes in the processes (black circles) and, also, about the significance (color changes from blue to red).

**Figure 2:**
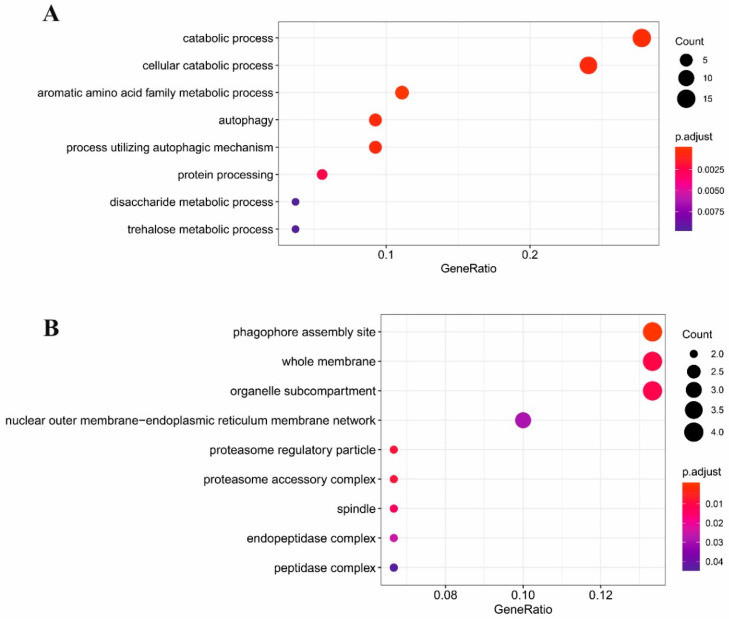
Results of GO GSEA of *P. rubens* downregulated genes for different GO domains’ (**A**) biological processes and (**B**) cellular components. No GO molecular functions were detected. The significance, as well as the rate of involvement of downregulated genes, are marked with the same colors and shapes.

To examine the functions of significantly expressed genes (1350 upregulated and 765 downregulated), we performed gene ontology by gene set enrichment analysis (GSEA). In order to classify genes into functional categories, gene ontology-based overrepresentation analyses were executed. We identified our dataset overrepresented in several biological processes (Figure 1A and Figure 2A), cellular components (Figure 1B and Figure 2B) and molecular functions (Figure 1C).

### 2.4. Functional Classes Analysis

**Figure 3:**
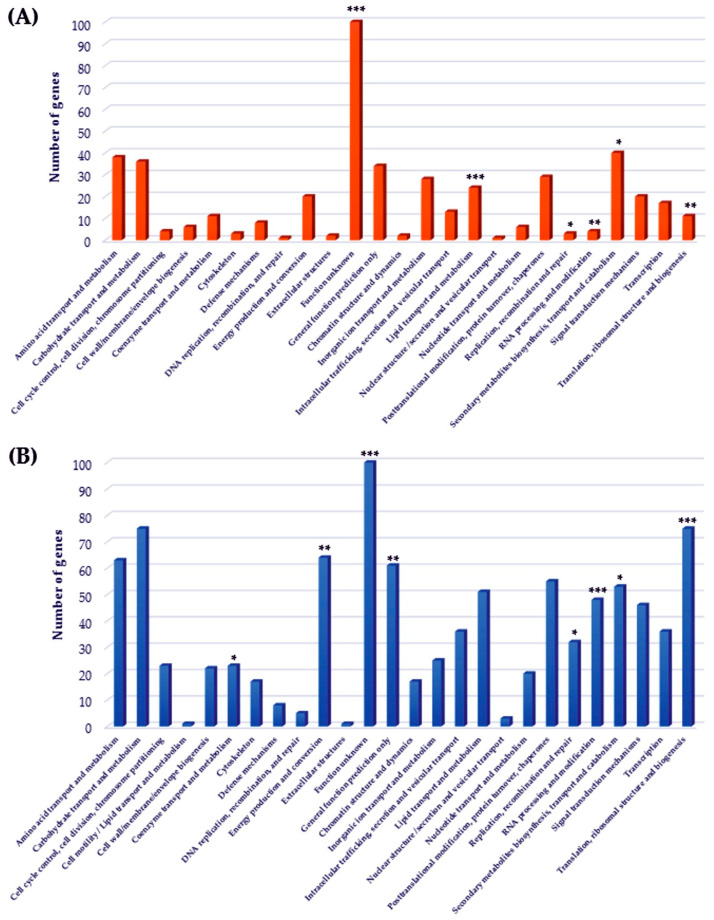
The most significant pathways affected by vapors of eight different EOs. Based on the results of the GO enrichment analysis, data shows changed expression level of genes (**A**) underexpressed and (**B**) overexpressed. * *p*-value ≤ 0.05, ** *p*-value ≤ 0.01 and *** *p*-value ≤ 0.001.

To interpret the data closer, we used overlapped the functional classification from the COG, KOG and KEGG BRITE databases [31]; however, many of the *P. rubens* genes were not well-characterized or with unknown functions. Genes with significantly changed expressions were classified. The occurrence of up- or downregulated genes in appropriate functional classes were compared to the overall gene representation in functional classes from the whole genome of the fungus presented in a microarray. We identified significantly dysregulated functional classes (Figure 3), where the increased occurrence of up- or downregulated genes was revealed. Figure 3 depicts the most significantly altered functional classes with significantly increased occurrences of upregulated genes: translation, ribosomal structure and biogenesis (*p* = 0.0000018); RNA processing and modification (*p* = 0.0001) and replication, recombination and repair (*p* = 0.01). The observed upregulation is moreover supported, with significantly less than the expected occurrence of downregulated genes in these classes.

Other significantly altered functional classes with statistically significant increases of upregulated genes were: energy production and conversion (*p* = 0.0056) and coenzyme transport and metabolism (*p* = 0.047) and, with the increase of downregulated genes, inorganic ion transport and metabolism (*p* = 0.0007) and secondary metabolites biosynthesis, transport and catabolism (*p* = 0.04). Significant increases in the occurrences of downregulated genes in the functions of unknown classes (*p* = 0.001) point to a number of genes that may play important roles in the processes of the response of *P. rubens* to EOs exposure. Unfortunately, their functions still remain unknown.

### 2.5. Metabolic Pathway Analysis

In the present study, considerable alterations in gene expression levels to stress responses on EOs vapors were found in *P. rubens*. We have been identified 99 altered pathways, containing 351 genes from our dataset (Figure S1). The occurrence of genes from our dataset was, together, 534, caused by the occurrence of some genes in more than one metabolic pathway. Commonly, we observed significant alterations of gene expressions in various metabolic pathways: nucleotide transport and metabolism; translation and protein biosynthesis processes; amino acid metabolism; metabolism of cofactor and vitamins; basic cellular processes; basic metabolism pathway and energy production and others (Table S2).

#### 2.5.1. Genes Involved in Protein Synthesis

In order to deeply understand the metabolic pathways of these differentially expressed genes, they have been grouped and systematically studied. Data analysis revealed that around 28% of genes are associated with the biosynthesis of proteins. The representation of genes that encode enzymes involved in amino acid (AA) metabolisms was strongly diverse. Enzymes coding aminoacyl-tRNA synthetases (aaRS) are located in the cytoplasm of the cell. Among these enzymes, tryptophanyl (Pc12g04630), lysyl (Pc12g09250), asparaginyl (Pc12g15910), methionyl (Pc16g02040), prolyl (Pc16g07440) and histidyl (Pc22g02880) were overexpressed compared to valyl (Pc20g09480), which had decreased expression.

In many pathways, like in β-lactam antibiotic production, analysis revealed changes on the transcriptional level in metabolic pathways, where AAs have a key role. The upregulated Pc20g04020 gene encodes a threonine synthase, an enzyme that is involved in threonine biosynthesis. The other gene, which showed a higher expression level, was Pc13g07730. This overexpressed gene is annotated as L-threonine ammonia-lyase. Results showed that 5-aminolevulinate synthase (Pc22g13500) is another upregulated protein involved in these biosynthetic pathways. In contrast, cystathionine gamma-synthase (Pc20g08350) showed a decreased transcriptional level over the aforementioned genes. Taken together, all these data and the other in Table S2, interference with the functioning of the cell is confirmed at the proteomic level.

#### 2.5.2. Transcriptional Modifications of Genes Encoding Carbohydrates

Our results showed that the metabolism of carbohydrates during the exposition of EOs is generally upregulated. From a range of metabolic pathways, encoded by multiple gene clusters, increased transcriptional levels were achieved: at glycolysis (Pc18g01490, Pc12g10630, Pc20g01630, Pc12g13500, Pc20g04410 and Pc12g16040); the pentose phosphate pathway (Pc12g02790, Pc13g14570, Pc12g13500 and Pc20g04410); fructose and mannose metabolisms (Pc16g12970, Pc22g09390, Pc21g05470, Pc20g01550, Pc13g12020, Pc12g09190, Pc12g13500, Pc21g04410, Pc21g04400 and Pc16g10970) and galactose metabolism (Pc12g13500, Pc14g00310, Pc12g07810 and Pc20g04410). The dominant gene in each group is 6-phosphofructokinase (Pc12g13500). It is responsible for transferring phosphorus-containing groups. In contrast, low levels of downregulated genes involved in the carbohydrate metabolism were obtained. As an example, Pc16g08460 the D-arabinitol dehydrogenase (NADP+) and Pc20g15580, the L-glyceraldehyde reductase. The list of all genes with changed transcript levels is displayed in Table S2.

#### 2.5.3. Changed Expression Level of Genes Involved in Fat Metabolism

The functional analysis of the genes revealed numerous altered metabolic pathways involved in lipid metabolism. Most of them remain with unknown functions that cannot be readily assigned. These hypothetical proteins have a certain similarity with conserve proteins that encode genes for fatty acids; steroid biosynthesis and glycerophospholipid, glycerolipid and sphingolipid metabolisms. Among them, Pc22g00420 is annotated as acetyl-CoA-acetyltransferase, also known as acetoacetyl-CoA thiolase, and demonstrated low levels of expression; it has a key role in the regulation of ergosterol synthesis. The other important protein is glycerol-3-phosphate O-acetyltransferase (Pc22g05820). An analysis revealed the overexpression of this gene. In addition to the above information, all alterations are summarized in Table S2.

#### 2.5.4. Gene Related to Secondary Metabolites

A gene-by-gene analysis showed that among the downregulated genes with hypothetical functions are just a few of them that are included in β-lactam resistance, aflatoxin biosynthesis or ABC transporters. The most interesting gene is pc18g01310, which codes for proteins regulating enzymatic reactions at the level of the cell wall and cellular organelles. It belongs to the group of chitin hydrolases -β-N-acetylhexosaminidase, which is released during autolysis into the medium. In total, two genes encoding enzymes involved in the biosynthesis of aflatoxin have shown reduced expression levels. The Pc12g16460 gene is linked by norsolorinic acid ketoreductase and pc22g19340-coding versiconal hemiacetal acetate esterase. In our investigation, EO treatments increased the expression level of the Pc21g18900 gene. It encodes a hypothetical protein included in monobactam biosynthesis and is named as the 4-hydroxy-tetrahydrodipicolinate synthase.

## 3. Discussion

In a fast-paced world, it is important to expand our knowledge and search for the undiscovered. The results of the present work demonstrate changes in the transcriptomic profile of the mold *P. rubens* in association with stress responses caused by the exposure to EO vapors. According to our literature research, there is no investigation studying the gene expression of *P. rubens* under exposure to the vapors of EOs.

Plant derivates, such as EOs, can modulate, inhibit and even kill the microorganism through specific mechanisms [32]. Unfortunately, the exact mechanism of EO effects is still not entirely clear; their different composition of substances makes them unique. The chemical compositions of assayed EOs are guaranteed by the producer based on the performed a GC/MS analysis and can be checked for each flask of EOs through the web page: https://sourcetoyou.com. EOs possess a wide spectrum of biological activities, including antimicrobial, antiviral, insecticidal and antifungal properties [33].

The regulation of genes responsible for protein synthesis is a limiting factor for cell survival. It can be inhibited in a number of ways, such as by altering the expression level of aminoacyl-tRNA synthetases [34]. In our case, treatments with EOs significantly show their power to decrease (for valyl-aaRS) or increase (tryptophanyl-, lysyl-, asparagynil-, methionyl-, prolyl- and histidyl-aaRS) the expression of several *P. rubens* genes. Modifications of these enzymes lead to changes in the mechanism of the translation of cellular proteins, metabolic and signaling pathways [34].

Amino acid metabolisms are an indispensable process in β-lactam production. Three AAs serve as building blocks, namely α-aminoadipate, L-cysteine and L-valine. A previous analysis demonstrated that the gene Pc20g04020 is considered to be unique for coding the threonine synthase in *P. rubens* [35]. It plays a key role in catalyzing the conversion of O-phospho-L-homoserine into L-threonine and phosphate. In the next step, L-threonine can be converted into another AA, such as L-cysteine. Pc13g07730 is annotated as L-threonine deaminase, which removes an amino group from L-threonine, resulting in the production of 2-oxobutanoate and pyruvate. The corresponding enzyme that catalyzes the conversion of glycine and succinal-CoA into 5-aminolevulinate, CoA and CO_2_ is 5-aminolevulinate synthase (Pc22g13500), also named as the pyridoxal 5’-phosphate-dependent (PLP) enzyme [35]. These findings suggested that the influence of applied EOs may play an important role in modulating these signaling pathways and, thus, may regulate processes involved in cysteine production, which is very important for beta lactam production [35].

According to current knowledge, gene Pc20g08350 is coding a cystathionine gamma synthase, which is a transsulfuration enzyme involved in the catalysis of the PLP-dependent γ-replacement of O-succinyl-L-homoserine and L-cysteine, yielding L-cystathione and acetate [35]. Hypothetically, the alteration of the β-lactam production can bring to the modification a synthesis of diverse compounds that can increase the vulnerability of the fungus against different environmental factors.

Carbohydrates have an irreplaceable role in fungi and represent an important part of the cell wall (glucans and chitin), as well in the role of storage polysaccharide (glycogen), disaccharide and sugar alcohols [36]. An interesting gene is 6-phosphofructokinase (Pc12g13500) which has a strong similarity to 6-phosphofructokinase pfkA to *Aspergillus niger* [37]. The Pc16g08460 gene is annotated as D-arabinitol dehydrogenase (NADP+) and has a low expression level. If compared with other fungi, this enzyme is considered as one of the few enzymes capable of utilizing arabitol as a main substrate [38]. The downregulated Pc20g15580 gene, coding L-glyceraldehyde reductase, takes part in a D-galacturonate degradation pathway and plays an important role in the pentose and glucuronate interconversion pathways [39].

The genus *Penicillium* includes species well-known for lipolytic enzyme productions, especially lipases and esterases, which are able to use lipids as carbon sources. They also participate as biocatalysts, which provide the hydrolysis of water-soluble short acyl chain esters as sure as water-insoluble long-chain triacylglycerols [40]. Acetyl-CoA-acetyltransferase (Pc22g00420) has a key role in the regulation of ergosterol synthesis and is the first main catalytic enzyme in the mevalonate pathway. This enzyme regulates the transformation of acetotyl-CoA from two molecules of acetyl-CoA [41]. If the ergosterol production is broken down, it leads to inhibition of the fungus growth. The biosynthesis of triglyceride is necessary to provide the decisive energy molecules, as well as in the biosynthesis of fatty acids and phospholipids [42]. Triglycerol-3-phosphate O-acetyltransferase (Pc22g05820) is an integral component of the membrane that has transferase activity. This upregulated enzyme is the first enzyme catalyzing the acylation of glycerol 3-phosphate in the glycerolipid metabolism [43].

Over the past decades, scientists have further explored the role of enzymes in biological processes [44], performing a wide genome sequencing of the filamentous fungus *P. chrysogenum* Wisconsin 54-1255. A transcriptomic analysis demonstrated the altered transcript levels in many metabolic pathways. Some of our outcomes coincided to their published results. The downregulated gene associated with the secondary metabolite productions was the glycoside hydrolases (Pc18g01310), also named as β-N-acetylhexosaminidase, which has a strong similarity to the hypothetical β-hexosaminidase A precursor in *Bacillus halodurans* [44]. This enzyme is an important part in the chitinolytic system in the cell wall of the growing fungus. It plays a key role in the controlling of the cell wall chitin lysis and, at the same time, in protecting cells from rupture [45].

The next affected upregulated gene is Pc21g18900, which belongs to the DapA family proteins, catalyzing the condensation of (S)-aspartate-beta-semialdehyde ((S)-ASA) and pyruvate to 4-hydroxy-tetrahydrodipicolinate (HTPA) [46]. It is located in the cytoplasm of the cell and has a predicted role in the biosynthesis of the secondary metabolites (monobactams) and lysine [47]. To date, the function of this enzyme is still not well-understood [48].

The study of the gene expression of *P. rubens* exposed to eight different EO vapors was never done before using a microarray system; moreover, we were able to get a big amount of data at once, which is currently almost unknown. This experiment can be considered as pioneering in understanding the effect of several EOs on various *P. rubens* biochemical pathways. The information obtained by this study allowed us to begin to comprehend the antifungal mechanisms of EOs in a more complete way.

## 4. Materials and Methods

### 4.1. Essential Oils

The study was performed using the following commercial EOs: arborvitae (TP) from *T. plicata* Donn., cassia (CC) from *C. cassia* L., clove (EC) from *E. caryophyllata* Thunb., lemongrass (CF) from *C. flexuosus* Nees ex Steud., melaleuca (MA) from *M. alternifolia* Maiden, Betche. oregano (OV) from *O. vulgare* L., peppermint (MP) from *M. piperita* L. and thyme (TV) from *T. vulgaris* L. (doTERRA, Pleasant Grove, UT, USA). In order to avoid photo-oxidation, the EOs were preserved in dark glass vials.

### 4.2. Fungal Strain and Fungicide Activity of the Vapor Phase of EOs

Antifungal activity of eight EOs were investigated against airborne mold *Penicillium rubens Wisconsin* 54-1255 (American Type Culture Collection-ATCC 28089). The fungus was cultivated on Malt Extract Agar (MEA; Sigma-Aldrich, Saint Louis, MO, USA) at 28 °C. In order to determine the fungicide activity of volatized EOs, a 5-mm square of growing fungal mycelia from MEA was placed into 5 mL of malt extract broth (MEB; Sigma-Aldrich) inside small (60-mm diameter) Petri dishes and incubated at room temperature (24–26 °C). Different quantity of EOs (Table 2) was applied on the inner surface of the Petri dish lid at dose levels of 1 µL/1 mL air space. As controls, served fungus *P. rubens* were placed into MEB medium without the application of EOs. To prevent vapor leakage, the Petri dishes were sealed with parafilm and incubated in the dark for 24 h for TP, CC, CF, OV, and TV; MP; EC and MA for 48 h at 22 °C. When the fungus reached the exponential phase, it was harvested. After cultivation, 50–100 mg of samples were collected by inoculation loop under aseptic conditions into sterile RNase-free plastic tubes, quickly frozen in liquid nitrogen and lysed in 1 mL of commercially available TRIzol^TM^ Reagent (Thermo Fisher Scientific, Waltham, MA, USA). Before storage at −80 °C or directly used to perform RNA isolation, the samples needed to be incubated for 30 min at room temperature.

### 4.3. RNA Isolation and Quality Control

Total RNA was isolated from fungal mycelia using a Direct-zol^TM^ RNA MiniPrep Plus (Zymo Research, Irvine, CA, USA) kit according to the protocol of the manufacturer. RNA quality was evaluated using the Experion Automated Electrophoresis System for RNA analysis (Bio-Rad Laboratories, Inc., Hercules, CA, USA), and the RNA concentration was measured using Nanodrop ND-2000 (Thermo Fisher Scientific). Total RNA degradation/quality was determined based on the 18S and 28S rRNA ratio. RNA samples with the RQI number (calculated based on the 18S/28S rRNA ratio) above the value 7.5 were selected for subsequent gene expression analysis.

### 4.4. Microarray Analysis of Gene Expression

Microarray analysis was performed using the total RNA and comparing the gene expression of the control sample with the samples treated with EOs. One-hundred nanograms of total RNA were amplified and labeled with a Low Input Quick Amp Labeling Kit (Agilent, Santa Clara, CA, USA) according to the manufacturer`s instructions. After, labeling samples were purified using a GeneJET RNA Purification Kit (Thermo Fisher Scientific) to remove unincorporated nucleotides. Three-hundred nanograms of labeled test samples vs. control samples were mixed and applied onto a *Penicillium rubens* Gene Expression 8x15K custom microarray slide (Agilent Technologies) and hybridized 17 h at 65 °C by rotating the slide at the speed 10 rpm in a hybridization oven (Agilent Technologies). After hybridization, two wash steps were performed (Gene Expression Wash Buffer Kit; Agilent Technologies), and the slide was scanned at a resolution of 2 µm using an Agilent Microarray Scanner.

### 4.5. Image and Data Analysis

Tagged Image File Format (TIFF) multiscan image was converted and processed using Feature Extraction Software 12.1 (Agilent Technologies) to acquire spot intensities. Acquired data were analyzed in GeneSpring 12.6 GX software to obtain differences in the gene expression. Significant differences in the fold of the gene expression change between the control and treated samples were ≥2.0. Finally, a pathway analysis was performed to reveal the molecular pathways significantly modified in our experiment (*p* ≤ 0.05). The R programming language version 3.3.3 [49] and the openxlsx library (github) were used. To perform a KEGG enrichment analysis, the Bioconductor library clusterProfiler [50] was applied to genes with a *p*-value cutoff 0.05 and BH *p*-value adjustment method (Benjamini – Hochberg method) [51]. Heatmaps from the KEGG enrichment analysis were created by Bioconductor libraries complexHeatmap [52] and circlize [53]. Biochemical pathways’ graphical representations were obtained using the pathview [54] library from Bioconductor.

## 5. Conclusions

In summary, the gene expression data obtained in this study reported the transcriptional changes in *P. rubens* genes influenced by the vapors of EOs. The study provides important findings regarding the understanding of the impact of EOs to fungal cells. The effects of EOs create a cascade of events that affect the metabolism and, consequently, the functions and structure of the fungal cell. It is evident that all the metabolic pathways, especially for vital functions such as polysaccharide and carbohydrate metabolisms, fatty acid metabolism, nucleotide and nucleoside metabolisms or the regulation of the production of SM, are deeply influenced by the antifungal properties of these natural compounds.

Each EO has different characteristics, and our study evidenced how some of them have more efficient antifungal activities and some others possess only a growth-inhibiting effect. Therefore, their application depends on the result that we wish to obtain, to kill or to mitigate. As natural extracts, they should be safe and can replace some more dangerous synthetic chemicals in order to disinfect indoor contaminated air. We have achieved precious experiences in the development and optimization of the microarray analysis, so it would be worth utilizing it in order to evaluate the antifungal properties of EO vapors against other indoor airborne fungi and compare the new data with this one.

## Figures and Tables

**Table 1:** The number of up- or downregulated genes presented in at least half of the samples after exposure to selected essential oils (EOs).

Essential Oil	No. Upregulated	No. Downregulated	No. of Genes Detected in at Least 4 Assayed EOs (*n* ≥ 4)
*Thuja plicata*	1730	1477	**1430** upregulated **833** downregulated
*Cinnamomum cassia*	1760	835	
*Eugenia caryophyllata*	1818	1728	
*Cymbopogon flexuosus*	1831	1043	
*Melaleuca alternifolia*	1121	702	
*Origanum vulgare*	1609	1221	
*Mentha piperita*	2419	1684	
*Thymus vulgaris*	1520	1004	

**Table 2:** Used essential oils their highest noncytotoxic concentrations.

Essential Oil	Concentration
*Thuja plicata (TP)*	0.025 µg/mL
*Cinnamomum cassia (CC)*	0.05 µg/mL
*Eugenia caryophyllata (EC)*	0.05 µg/mL
*Cymbopogon flexuosus (CF)*	0.025 µg/mL
*Melaleuca alternifolia (MA)*	0.05 µg/mL
*Origanum vulgare (OV)*	0.01 µg/mL
*Mentha piperita (MP)*	0.025 µg/mL
*Thymus vulgaris (TV)*	0.05 µg/mL

## Supplementary Materials

The following are available online at https://www.mdpi.com/2079-6382/9/6/343/s1: Figure S1. Hierarchical clustering of differentially expressed genes, illustrated by the heatmap. Table S1. List of regulated genes. Table S2: List of genes and their corresponding metabolic pathways.
