# Supplementary material for: Studying the Gene Expression of Penicillium rubens Under the Effect of Eight Essential Oils
Source: Antibiotics (Basel). 2020 Jun 19;9(6):343. doi: 10.3390/antibiotics9060343 (PMC7345231; doi:10.3390/antibiotics9060343)
Supplement: Supplementary file 1 [file antibiotics-09-00343-s001.zip › Supplementary_Files/Captions_supplementary.pdf]

Supplementary Material

Studying the gene expression of *Penicillium rubens* under the effect of eight essential oils

Zuzana Kisová[1], Andrea Šoltýsová[2,3], Mária Bučková[1], Gábor Beke[1], Andrea Puškárová[1], Domenico Pangallo[1,*]

[1]Institute of Molecular Biology, Slovak Academy of Sciences, Dúbravská cesta 21, 84551 Bratislava, Slovakia.

[2]Faculty of Natural Sciences of Comenius University, Department of Molecular Biology, Ilkovičova 6, 84215 Bratislava, Slovakia.

[3]Institute for Clinical and Translational Research, Biomedical Research Center, Slovak Academy of Sciences, Dubravska cesta 9, 84505 Bratislava, Slovakia

*Corresponding author: domenico.pangallo@savba.sk

Fig. S1. Hierarchical clustering of differentially expressed genes, illustrated by the heatmap. From 351 of genes, 74 showed low expression (red color) compared to 277 with up-regulated genes (green color).

Table S1. List of regulated genes.

Table S2. List of genes and their corresponding metabolic pathways.
